# Reversibility of a Point Mutation Induced Domain Shift: Expanding the Conformational Space of a Sucrose Phosphorylase

**DOI:** 10.1038/s41598-018-28802-2

**Published:** 2018-07-11

**Authors:** Michael Kraus, Clemens Grimm, Jürgen Seibel

**Affiliations:** 1 Institute of Organic Chemistry, University of Würzburg, Am Hubland, 97074 Würzburg, Germany; 2 Department of Biochemistry, Theodor Boveri-Institute, University of Würzburg, Am Hubland, 97074 Würzburg, Germany

## Abstract

Despite their popularity as enzyme engineering targets structural information about Sucrose Phosphorylases remains scarce. We recently clarified that the Q345F variant of *Bifidobacterium adolescentis* Sucrose Phosphorylase is able to accept large polyphenolic substrates like resveratrol via a domain shift. Here we present a crystal structure of this variant in a conformation suitable for the accommodation of the donor substrate sucrose in excellent agreement with the wild type structure. Remarkably, this conformation does not feature the previously observed domain shift which is therefore reversible and part of a dynamic process rather than a static phenomenon. This crystallographic snapshot completes our understanding of the catalytic cycle of this useful variant and will allow for a more rational design of further generations of Sucrose Phosphorylase variants.

## Introduction

Sucrose Phosphorylases (CAZy Family GH 13^1^, SPs) are popular targets for enzyme engineering and employed in various transglucosylation reactions^2,3^ because they utilize the cheap and abundant donor substrate sucrose^4^, exhibit thermostability^3^ and organic solvent compatibility^3,5^. Several SP variants have been created in recent years in order to establish a variety of novel transglucosylation reactions^6–9^. While the native reaction of SPs is the interconversion of sucrose and α-d-glucose-1-phosphate via a covalent enzyme-glycosyl intermediate^10^, the two main target reactions for enzyme design are the synthesis of rare disaccharides^7,8^ and the glucosylation of polyphenols^6,9,11^ Several crystal structures of *Bifidobacterium adolescentis* Sucrose Phosphorylase^10,12^ have elucidated the catalytic mechanism and substrate binding of wild type SPs^10^ and constitute a common starting point of engineering strategies.

The most crucial insight into the mechanism of SPs was gained in 2006 when Miza *et*. *al*. revealed the existence of two distinct conformations of BaSP, one responsible for the accommodation of sucrose, the other for α-d-glucose-1-phosphate^10^. BaSp switches between those two conformations via the rearrangement of two flexible loops: Loop A (^336^AAASNLDLY^344^, part of domain B’) and loop B (^132^YRPRP^136^, part of domain B). Of note, the invariant residue Gln345 targeted in this study borders loop A but maintains an identical position in both loop conformations. In the sucrose binding conformation (2gdu, 1r7a, 2gdv Chain A) loop A points into the active site and Asp342 becomes part of the acceptor binding site forming a H-bond to OH-3 of the fructosyl moiety. The sidechain of Tyr344 faces away from the +1 site and is not involved in substrate binding.

The proposed phosphate binding conformation^10^ (2gdv, Chain B, features loop A facing away from the active site, while the sidechain of Tyr344 now points into it and contributes to solvent shielding. The change in loop B mostly consists in the rearrangement of Arg135 which is now oriented towards the catalytic centre and facilitates phosphate binding through its positive charge^10^ Neither conformation features a defined access channel, consequently, access via substrate diffusion must occur by one or more unknown open conformations.

**Figure 1:**
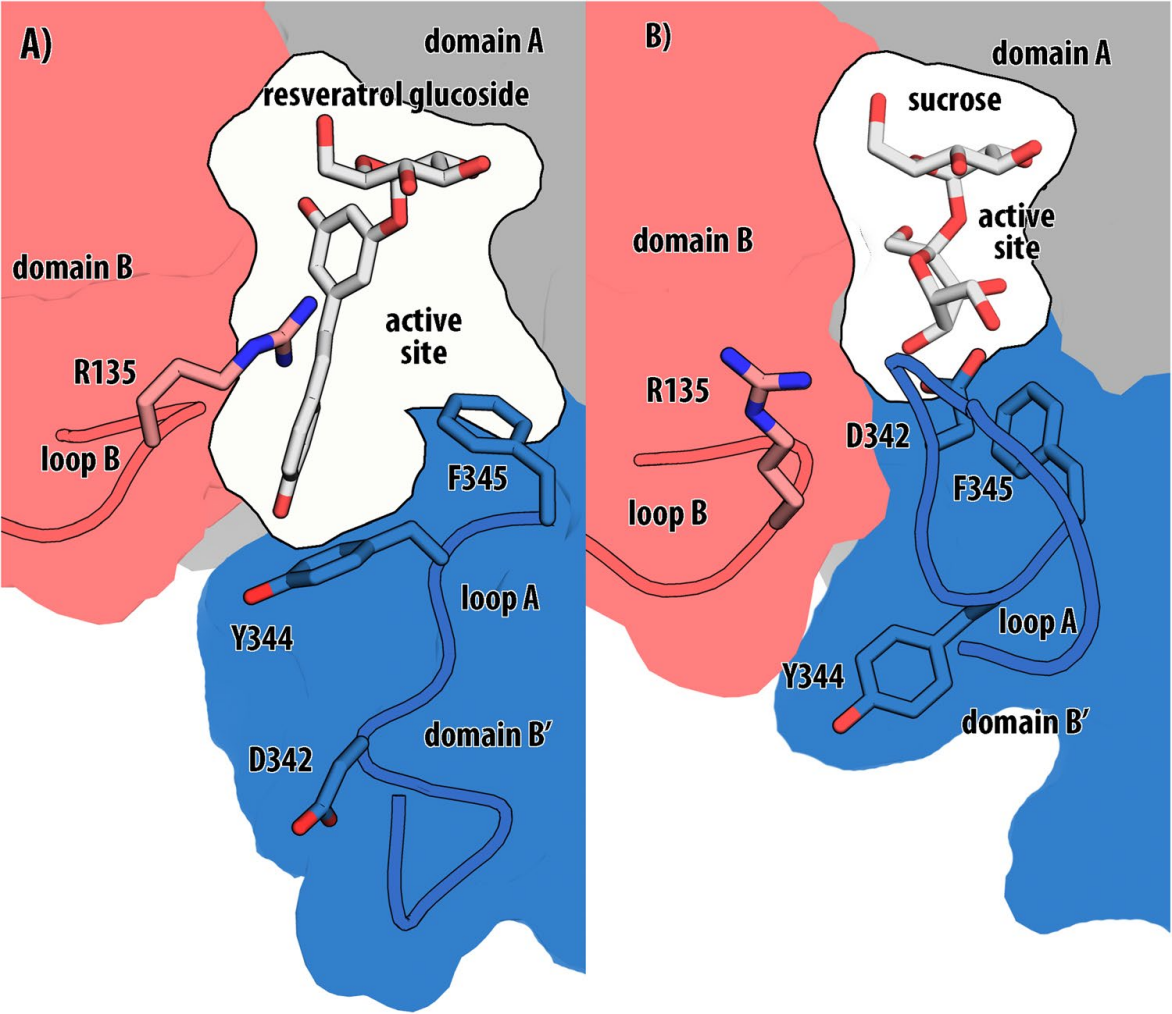
Schematic representation of BaSP Q345F loop conformations. Blue: Domain B’, Red: Domain B, Grey: Domain A, White: active site cavity. (**A**) Aromatic compound binding conformation of BaSP Q345F (in complex with resveratrol-α-D-glucoside (PDB ID code 5man)). (**B**) Sucrose binding conformation of BaSP Q345F, sucrose superimposed from 2 gdu). It should be noted that the increase in acceptor site space is due to the domain shift and not a result of the loop rearrangement.

The first structural insight into the mechanism of sucrose phosphorylase variant Q345F was presented recently^9,11^. The BaSP Q345F variant features a loop orientation that resembles the wildtype phosphate binding conformation (Fig. 1). The two key differences between the wildtype and variant crystal structures are (1): the orientation of Tyr344, which is not part of the active site, and (2): a movement of the entire domain B by 3.3 Å^9,11^ referred to as a domain shift. We recently demonstrated that this domain shift is in fact responsible for the altered acceptor specificity spectrum that enabled the synthesis reaction of resveratrol-3-α-d-glucoside and nigerose^8,9,11^ While we were able to demonstrate that the domain shift is ligand independent^11^, the question of how the variant binds sucrose and whether the domain shift is static and permanently present in the Q345F variant remained unsolved. We now present the missing link, a crystal structure of BaSP Q345F in the sucrose binding conformation. Based on this structural evidence the full catalytic cycle of this BaSP variant is now understood and can now be targeted by further design studies.

## Results

**Figure 2:**
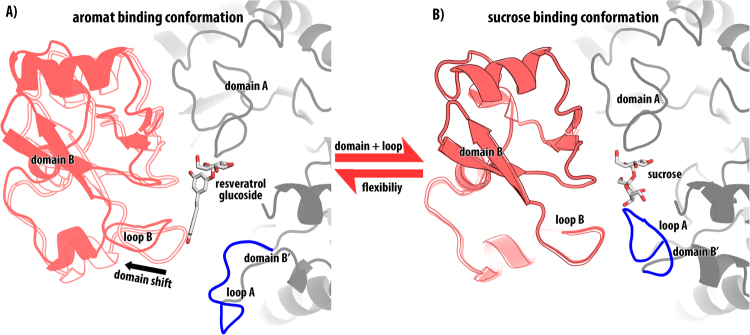
Reversible domain shift induced by the Q345F exchange. The domain shift occurs together with the rearrangement of loop A (blue). (**A**) Aromatic compound binding conformation of BaSP Q345 F in complex with the resveratrol-3-α-d-glucosid. Domain B (red) shifts by 3 Å (red outlines). (**B**) Sucrose binding conformation of BaSP Q345F doamin B (red) occupies the same condition as found in the wildtype (red outline). The two crystall structures likely represent the two extremes of a dynamic equilibrium.

**Figure 3:**
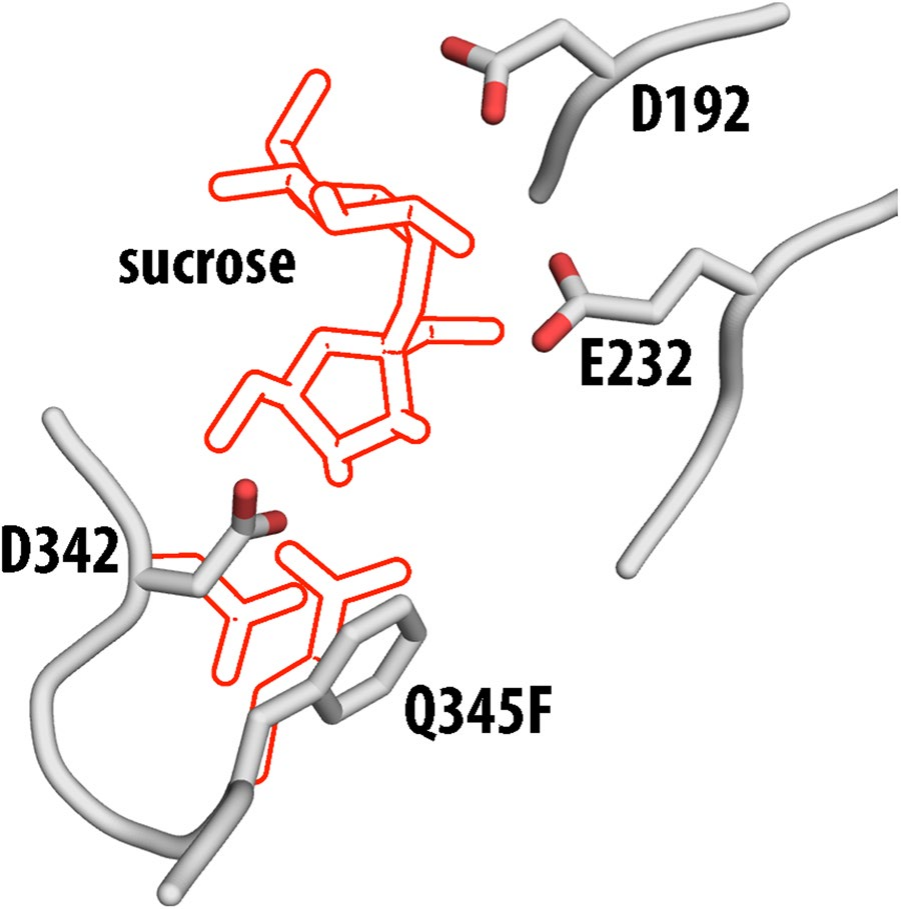
Key active site residues of BaSP Q345F in the sucrose binding conformations. The outlined sidechaines represent the key differences between the wild type and the variant: The rotation of D342 and the Q345F exchange. The position of sucrose from the aligned wild type structure is indicated as outlines as well.

In contrast to all previous structures of the Q345F variant, no domain shift is observed in the new crystal structure presented here. This shows that the domain shift is reversible and part of a dynamic process. (Fig. 2) The orientation of residue Asp342 presents the sole significant difference to wildtype BaSP (Fig. 3). This residue usually interacts with the 4-OH group of fructose and is rotated by 81° towards the −1 site relative to its orientation found in the wild type enzyme. The phenyl ring of Phe345 is rotated by 31° around the Cβ-Cγ axis relative to the amide of Gln345. During the loop rearrangement and domain shift the benzene ring of Phe345 rotates by 82°. This rotation is observed in all structures that display the domain shift, regardless which, if any ligands are present and causes the displacement of the neighbouring Tyr344 which is also linked to the domain shift.

**Figure 4:**
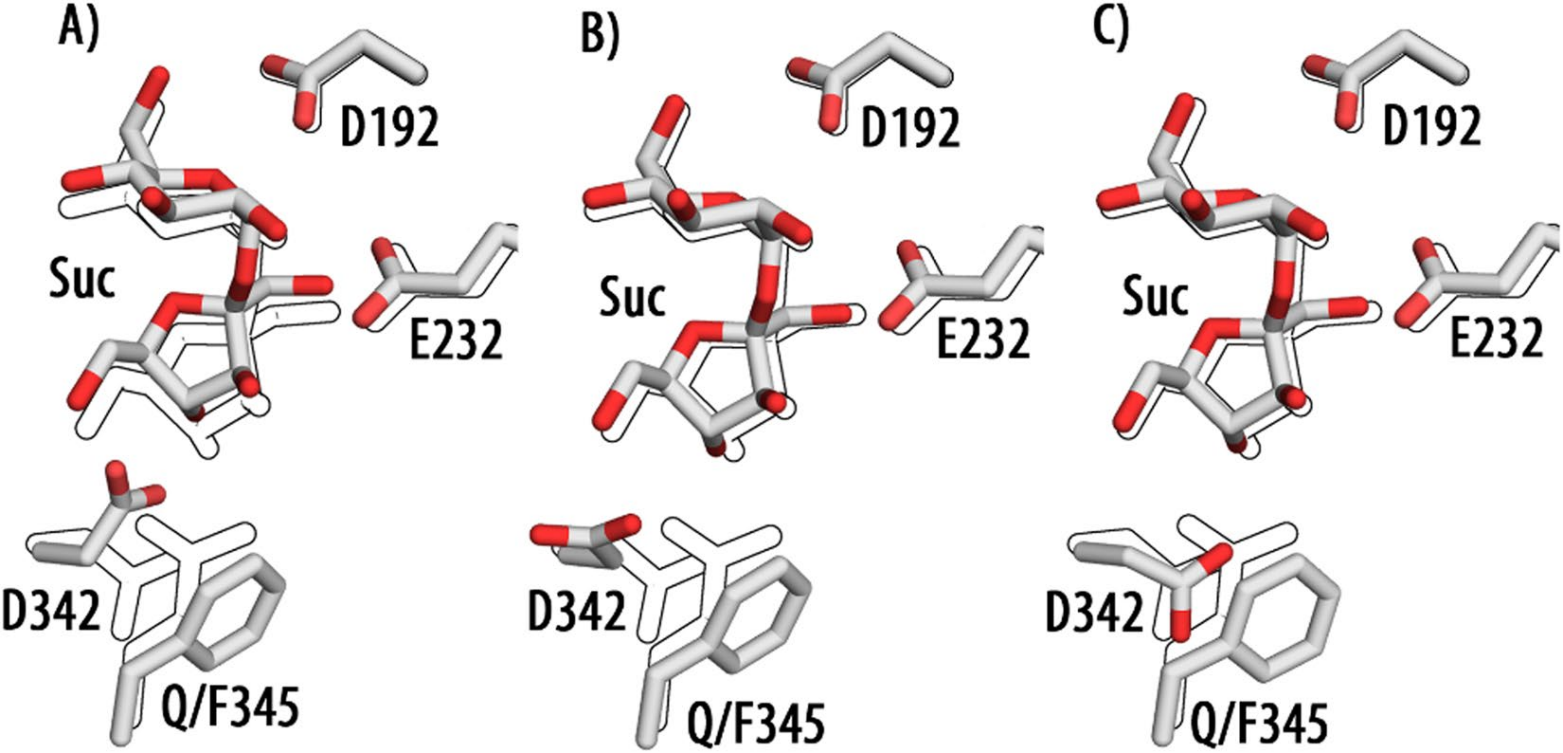
Orientation of sucrose and key side chains in BaSP Q345F as calculated by AutodockVina The outlines indicate the orientation of sucrose and the amino acid in the wild type (PDB ID 2gdu) (**A**) all side chains rigid (**B**) Asp342 defined as flexible (**C**) Asp342 and Phe345 defined as flexible.

The BaSP Q345F variant displays a lowered affinity for (K_M_ = 5.41 mM vs 0.91 mm wildtype) and activity towards (8.6% of the wildtype) sucrose^9,11^. This was to some degree expected as Gln345 exhibits hydrogen bonds with OH-3 and OH-6 of the fructosyl moiety of sucrose^10^. To evaluate the impact of the Q345F exchange and the rotation of Asp342 on sucrose binding docking studies with Autodock Vina were conducted. Docking of sucrose into BaSP Q345F yields a relative binding energy that is 10.1 kcal lower than the one of the wild type. This is due to the orientation of Asp342, which clashes with OH-4 and OH-6 of fructose and prevents sucrose from assuming the correct position in the enzyme (Fig. 4A). When Asp342 is defined as flexible the docking results show it facing away from its natural position by 88° (Fig. 4B). This does not recover its native H-bond but removes the steric clash and the difference in affinity to the wildtype is reduced to 2.0 kcal/mol. If Phe345 is set as a further flexible residue it rotates slightly and Asp342 can now adopt its native conformation and the total loss of affinity is reduced to 1.5 kcal/mol (Fig. 4C). The loss of the H-bonds between Gln345 and the fructosyl moiety were expected to reduce the affinity for sucrose. To examine this influence Gln345 was exchanged *in silico* against alanine to remove any interactions and docking with sucrose was performed. The result indicates that Gln345 contributes by ca. 0.9 kcal/mol to the donor binding. The remaining 0.6 kcal/mol difference is likely due to a slight steric hindrance induced by Pher345 (Table S2). It can be concluded that the Q345F exchange did not affect sucrose accommodation beyond the loss of the polar interactions of Gln345.

## Discussion

The previously observed domain shift is responsible for the ability of BaSP Q345F to glucosylate resveratrol and further polyphenols as well as synthesize nigerose^10^. The fact that the domain shift is absent in the sucrose binding conformation of BaSP Q345F indicates a reversible, dynamic process induced by the mutation. We conclude from the crystal structure presented here that BaSP Q345F exists as an equilibrium and the crystal strucutres represent the two endpoints of the process: The open conformation, which is required for the glycosylation of polyphenolic acceptors and the sucrose binding conformation which is needed for donor substrate conversion.

From these structures the catalytic mechanism of BaSP Q345F can be proposed: First sucrose enters BaSP Q345F via an open conformation which features the domain shift. The enzyme binds sucrose and the loop rearrangement occurs and simultaneously domain B shifts back into the wildtype-like position. The loss of H-bonds between Gln345 and the fructosyl moiety and a mior steic clash from Phe345 reduce the binding energy of sucrose, resulting in a lowered activity of the variant compared to the wild type otherwise the mutation does not affect sucrose recognition. Then fructose cleavage takes place in the sucrose binding conformation, which is identical for both wildtype and variant. Subsequently the loop rearrangement and the domain shift occurs again and BaSP Q345F transforms into a glucosyl-linked open conformation. A polyphenolic acceptor is recruited and after glucosyl transfer and product release the cycle begins anew. The reversibility of the domain shift is necessary for the efficient conversion of sucrose the domain shift itself for the ability to glucosylate the aromatic compounds. Further investigations into the mechanics of the domain movement, while beyond the scope of this publication, could lead to the ability to predict this phenomenom. A reversible domain shift may then constitute a powerfull novel tool for enzyme design as it allows to drastically alter the active site of a flexible enzyme, while maintaining the original structural features of the protein simulatiously.

## Conclusions

In summary the domain shift of BaSP Q345F introduced by the mutation is not a static effect but part of a dynamic process. Sucrose binding by BaSP Q345F takes place in a manner closely related to the wild type and the Q345F mutation has no effect in sucrose coordination aside from the predicted loss of the H-bonds between Gln345 and OH-3 and OH-6 of fructose. The existence of a BaSP Q345F conformation without the domain shift allows further interpretation of previous results. Now the complete set of conformations required for the catalytic cycle of BaSP Q345F are known and this information can be used for further engineering of the versatile Sucrose Phosphorylases and potentially be transferred to other members of the vast glycosdase family GH13.

## Methods

### Expression and Purification BaSP Q345F

As previously described^9^: *E*. *coli* Bl21 star™ cells were heat shock transformed with plasmid pET-28b(+)-BaSP-Q345F. Overnight cultures of the transformed host in LB-medium containing 50 mg/L kanamycin sulfate were grown and 1.8 mL was used to inoculate 250 mL of LB-medium (50 mg/L kanamycin sulfate). The cultures were incubated at 37 °C and 180 rpm until they reached an OD600 of 0.6, at which point the temperature was adjusted to 19 °C and IPTG was added to a final concentration of 0.5 mm. The cells were grown for additional 18 hours after which they were harvested by centrifugation (4000 g for 10 min). The sediment was resuspended in lysis buffer (60 mm phosphate, 250 mm NaCl, 11 mm imidazol). Cells were lysed using a sonicator and centrifuged at 17000 g for 10 min at 4 °C. The lysate was loaded onto 0.5 mL Ni-NTA columns equilibrated with lysis buffer and incubated at 4 °C and slow rotation for a minimum of 2 hours. The column was washed with 2.5 mL of lysis buffer and the protein was eluted with 1.5 mL of elution buffer (60 mm phosphate, 250 mm NaCl, 230 mm imidazol). The buffer was exchanged to 20 mm MOPS-NaOH-buffer (pH = 7) using 5 mL Hi-Trap columns from GE Healthcare.

### Crystallization, soaking data collection

Crystals were grown using the hanging drop method. 0.5 µl of 14 g/L Protein solution were mixed with 0.5 µl precipitant solution containing PEG 4000 (26–34% (w/v)), NaCl (200 mM) and Tris-HCl-buffer (pH = 7–9, 100 mM). Crystals were grown for 10 weeks at 16 °C up to a size of 0.05 × 0.04 × 0.08 mm. Crystals were then transferred to cryo solution containing PEG 1500 (30%(w/v)), glycerol (20%(w/v)) NaCl (200 mm) Tris-HCl-buffer (100 mm pH = 8) and plunged into liquid nitrogen. At beamline ID30B of the ESRF Grenoble the mounted crystals were placed within a 100 K nitrogen gas stream and datasets were collected over 180° oscillation range. The datasets were auto indexed, integrated and scaled with XDS.

### Structure determination and -refinement

The structures of BaSP Q345F solved by molecular replacement using chain B of PDB entry 2GDV as a search model within PHASER^13^. After initial refinement within Phenix^14^, regions with distinct conformational changes were manually rebuilt within COOT^15^ and the appropriate ligands were modelled into the active site. After three more rounds of automated refinement and manual rebuilding including water and ligand placement, the R and Rfree factors converged.

### Docking

The crystal structures of the BaSP E232Q (PDB ID 2gdu, chain A) and BaSP Q345F F-conformation (PDB 6FME) were used as “receptor” for the docking calculations. All water molecules and ligand entries were removed, non-polar hydrogens were added using AutoDockTools 1.5.6r^16^. For dockings with flexible residues the respective amino acids were defined as flexible and Gasteiger charges were added and rotatable bonds were assigned using AutoDockTools. The *in silico* mutations were introduced with pymol 1.8.0.3 using the mutagenesis wizard. Grid box center and grid dimensions (20 × 20 × 20 Å, grid spacing: 1.0 Å) were determined via AutoDockTools and transferred to the AutoDockVina configuration file.

As ligand sucrose as present in the crystal structure of BaSP E232Q was used. Gasteiger charges were added and rotatable bonds were assigned using AutoDockTools.

AutoDockVina^17^ was used for docking calculations. The docking parameters “exhaustiveness” and “energy_range” were set to “20” and “8”, respectively.

### Data availability

The corresponding coordinates and structure factors were submitted to the PDB under accession code 6FME. (They will become available upon publication). Other data generated or analysed during this study are included in this published article (and its Supplementary Information files).

## Electronic supplementary material

Supporting information
