# Supplementary material for: Reversibility of a Point Mutation Induced Domain Shift: Expanding the Conformational Space of a Sucrose Phosphorylase
Source: Sci Rep. 2018 Jul 11;8:10490. doi: 10.1038/s41598-018-28802-2 (PMC6041289; doi:10.1038/s41598-018-28802-2)
Supplement: Supplementary file 1 — Supporting information [file 41598_2018_28802_MOESM1_ESM.pdf]

Supplementary Information

Reversibility of a Point Mutation Induced Domain Shift: Expanding the Conformational Space of a Sucrose Phosphorylase

Michael Kraus, Clemens Grimm, Jürgen Seibel

Correspondence to: seibel@chemie.uni-wuerzburg

clemens.grimm@biozentrum.uni-wuerzburg.de

This PDF file includes:

Materials and Methods

Tables S1 to S2

Supplementary Information outline

1. Materials and Methods 2
1.1 Cloning expression and purification of BaSP Q345F 2
1.2 Construction of BaSP Q345F 2
1.3 SDS-PAGE of purified BaSP Q345F 2
1.4 Data collection and refinement statistics 3
1.5 Docking results 4
2 References 4

1. Materials and Methods

1.1 Cloning expression and purification of BaSP Q345F

As previously described[1]: Freeze-dried cultures of *B. adolescentis* (DSMZ 20083) were obtained from DSMZ (Deutsche Sammlung von Mikroorgansimen und Zellkulturen GmbH), and grown under anaerobic conditions in DSMZ medium Nr.58 without resazurin. Cells were harvested and the genomic DNA isolated, using a GenJet Genomic DNA purification Kit (Thermo Fisher). The BaSP gene was amplified from genomic DNA using the primers

5'-ATAACCATGGCTATGAAAAACAAGGTGCAGCTCATCAC-3' and 5'-CAATCCGCCTGTCGTCGCCCTCGAGTAAT-3'. The amplicon was inserted into pET-28b(+) using the NcoI and XhoI restriction sites yielding plasmid pET-28b(+)-BaSP-wt.

1.2 Construction of BaSP Q345F

As previously described[1]: The Q345F mutation was constructed applying the Megaprimer method. The mutagenic primers 5'-CCAATCTCGACCTCTACTTCGTCAACAGCACCTAC-3', and 5'-CAATCCGCCTGTCGTCGCCCTCGAGTAAT-3' were used for the creation of the megaprimer and 5'-ATAACCATGGCTATGAAAAACAAGGTGCAGCTCATCAC-3' was used for the second PCR. Cloning and purification of the variant followed the procedure described for the wildtype.

1.3 SDS-PAGE of purified BaSP Q345F

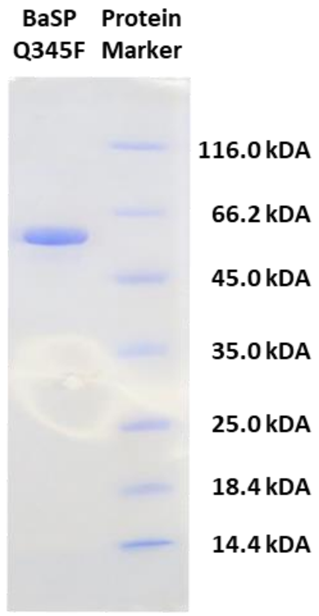

1.4 Data collection and refinement statistics

Table S1 Data collection and refinment statistics

Wavelength [Å]	*0.97625*
Resolution range	42.33 - 1.51
Space group	P 21 21 21
Unit cell (a, b, c, α, β, γ)[Å, Å, Å, °, °, °]	76.01 101.925 152.569 90 90 90
Total reflections	665032 (66346)
Unique reflections	175921 (17788)
Multiplicity	3.8 (3.7)
Completeness [%]	95.0 (97.0)
Mean I/sigma(I)	10.32 (0.89)
Wilson B-factor	17.78
R-merge	0.08066 (1.239)
Reflections used in refinement	175552 (17783)
R-work	0.1670 (0.3180)
R-free	0.1879 (0.3347)
Number of non-hydrogen atoms	9200
macromolecules	8002
Ligands	42
Solvent	1156
Protein residues	1011
RMS(bonds) [Å]	0.008
RMS(angles) [°]	1.07
Ramachandran favoured [%]	98
allowed [%]	1.9
outliers [%]	0.099
Clashscore	4.49
Average B-factor [$Å^2$]	23.45
Macromolecules	21.68
Ligands	14.53
Solvent	36.02

Statistics for the highest-resolution shell are shown in parentheses.

1.5 Docking results

Table S2 Docking of sucrose into BaSP wild type and BaSP Q345F as well as in-silico generated variants.

	flexible residues	*Relative Affinity in kcal/mol*
wild type (2gdu)	-	-12.8
Q345A (from 2gdu)	-	-11.9
D342A (from 2gdu)	-	-12.4
Q345F	-	-2.7
Q345F	D342	-10.8
Q345F	D342, Q345F	-11.3

2 References

1. M. Kraus, C. Grimm and J. Seibel, *ChemBioChem*, 2016, **17**, 33-36.
